# Supplementary figures and images for: Effectiveness of different central venous catheter fixation suture techniques: An in vitro crossover study
Source: PLoS One. 2019 Sep 12;14(9):e0222463. doi: 10.1371/journal.pone.0222463 (PMC6742355; doi:10.1371/journal.pone.0222463)

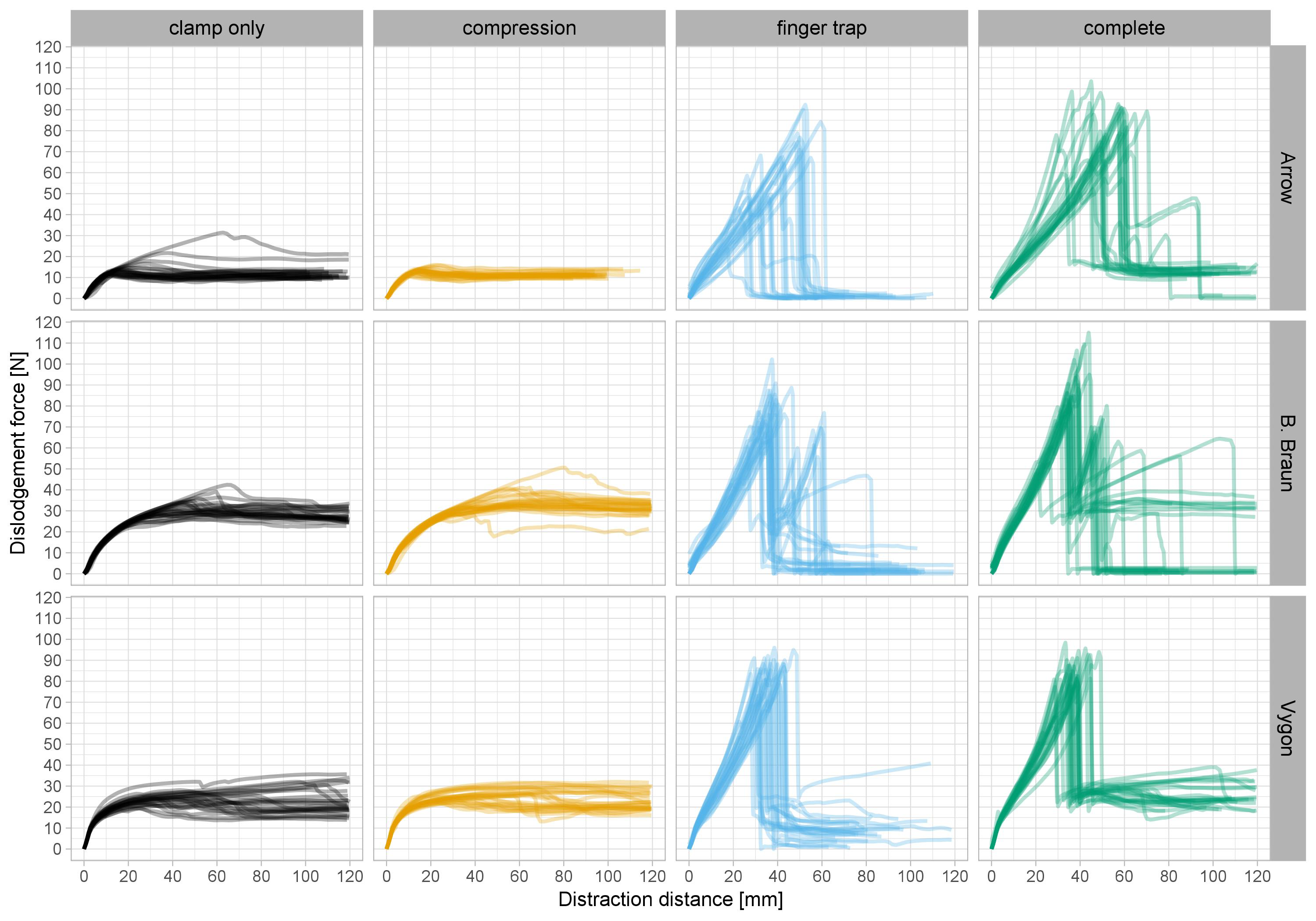

Supplement: S1 Fig — (TIFF) [file pone.0222463.s002.tiff]
