# Supplementary material for: Effectiveness of different central venous catheter fixation suture techniques: An in vitro crossover study
Source: PLoS One. 2019 Sep 12;14(9):e0222463. doi: 10.1371/journal.pone.0222463 (PMC6742355; doi:10.1371/journal.pone.0222463)
Supplement: Attachment — Submitted filename: CVC_Sutures_RESPONSE_05082019.doc [file pone.0222463.s003.doc]

**Response to reviewers comments**

Reviewer #1: The authors present an in vitro study comparing the effects of four suturing techniques on maximum dislodgment forces in CVCs from three different manufacturers. The authors conclude that the „finger trap“ fixation needs the highest force to dislodge the CVC.

* The methods section should be condensed. The first sentence (p 4, l 67) can be discarded since it just repeats the last sentence of the introduction. Most of the sub-headers can be removed. The information written in the paragraph for ‚test setting‘ can be mentioned in other parts of the methods.

RESPONSE:

We would like to thank the reviewer for his comments. We have changed all items as suggested.

* The results state a lot of significances but it is not said what these significance stay for. Please, rather state the difference of the statistical significant findings (I.e. high or lower dislodgment rate).

RESPONSE.

We have completely rewritten this paragraph for clarity. The results section now provides detailed information which dislodgement forces were higher or lower, e.g. “finger trap” and “complete” also resulted in higher dislodgement forces than the “compression” technique.

* Is Fig. 5 really important to understand the manuscript? It is only mentioned with one sentence in the results without explaining what this figure is telling us. Please, elaborate or discard.

RESPONSE:

We have added some more linking information regarding Fig. 5. From our point of view, Fig. 5 is essential for the manuscript because it presents the detailed dislodgement forces for all four fixation techniques and all three manufacturers. Utilizing Fig. 5, the reader can easily see that dislodgement forces are tremendously higher using the “finger trap” or “complete” approach compared to “clamp only” or “compression”. Thus, Fig. 5 helps to demonstrate the benefit of “finger-trap” based approaches at a glance.

* The authors write in their conclusion that the combination of the techniques “clamp only” plus “finger trap” might be a reasonable alternative. Although it is reasonable to discuss this it should not be mentioned in the conclusion since this combination was not tested in the study.
RESPONSE:

We have removed this information from the conclusion part and provided it in the discussion part instead.

\## Minor comments
* Protected names should be marked with a trademark sign

RESPONSE:

We have provided all protected brand names either with ® or ™ signs due to the information provided by the manufacturers.

Furthermore, we have added in the Methods section: “For the sake of simplicity, the terms “Arrow”, “B. Braun” and “Vygon” will be used consistently below.”

* page 4, lines 61-64: in an in vitro study ‚aim‘ instead of ‚outcome‘ would be the more suitable term.

RESPONSE:

We have changed this term as suggested.

* page 7, line 139: ratios are usually given as part of 1 instead of percentage. It is not clear what kind of ratio you mean (what is your reference of the ratio?)

RESPONSE:

We have changed this part as suggested. The information is now presented more precisely and percentages have been replaced by parts of 1.

* Please, add number of measurements for each column as well as results of statistical tests in Table 1

RESPONSE:

We have now provided the number of measurements for each columns and the statistical results in the legend of Table 1.

We would like to thank the reviewer again for his constructive comments and hope that we have addressed all items appropriately.

We have added another supplementary file including the raw data of our measurements (S1_Dataset) in order to comply with the Journals data sharing policy.
